# The velocity of the arterial pulse wave: a viscous-fluid shock wave in an elastic tube

**DOI:** 10.1186/1742-4682-5-15

**Published:** 2008-07-29

**Authors:** Page R Painter

**Affiliations:** 1 Office of Environmental Health Hazard Assessment, California Environmental Protection Agency, P. O. Box 4010, Sacramento, CA 95812, USA

## Abstract

**Background:**

The arterial pulse is a viscous-fluid shock wave that is initiated by blood ejected from the heart. This wave travels away from the heart at a speed termed the pulse wave velocity (PWV). The PWV increases during the course of a number of diseases, and this increase is often attributed to arterial stiffness. As the pulse wave approaches a point in an artery, the pressure rises as does the pressure gradient. This pressure gradient increases the rate of blood flow ahead of the wave. The rate of blood flow ahead of the wave decreases with distance because the pressure gradient also decreases with distance ahead of the wave. Consequently, the amount of blood per unit length in a segment of an artery increases ahead of the wave, and this increase stretches the wall of the artery. As a result, the tension in the wall increases, and this results in an increase in the pressure of blood in the artery.

**Methods:**

An expression for the PWV is derived from an equation describing the flow-pressure coupling (FPC) for a pulse wave in an incompressible, viscous fluid in an elastic tube. The initial increase in force of the fluid in the tube is described by an increasing exponential function of time. The relationship between force gradient and fluid flow is approximated by an expression known to hold for a rigid tube.

**Results:**

For large arteries, the PWV derived by this method agrees with the Korteweg-Moens equation for the PWV in a non-viscous fluid. For small arteries, the PWV is approximately proportional to the Korteweg-Moens velocity divided by the radius of the artery. The PWV in small arteries is also predicted to increase when the specific rate of increase in pressure as a function of time decreases. This rate decreases with increasing myocardial ischemia, suggesting an explanation for the observation that an increase in the PWV is a predictor of future myocardial infarction. The derivation of the equation for the PWV that has been used for more than fifty years is analyzed and shown to yield predictions that do not appear to be correct.

**Conclusion:**

Contrary to the theory used for more than fifty years to predict the PWV, it speeds up as arteries become smaller and smaller. Furthermore, an increase in the PWV in some cases may be due to decreasing force of myocardial contraction rather than arterial stiffness.

## Introduction

Following the opening of the aortic valve in early systole, blood pressure in the aorta rises rapidly as does the velocity of blood flow. This increase in blood pressure and momentum travels the length of the aorta and is also passed on to blood in arteries that branch from the aorta (e.g. carotid, brachial and mesenteric arteries). The phenomenon of rapidly increasing pressure and velocity spreading from the aortic root to distal arteries is termed the pulse wave.

The pulse wave is an example of a traveling wave in a fluid. Other examples are tsunamis and sound waves including sonic booms. In each of these, momentum moving in the direction of the wave increases pressure ahead of the wave's peak, and this increased pressure increases momentum ahead of the wave's peak. The speed at which the peak of a shock wave moves depends on physical properties of the fluid, dimensions of the space that bounds the fluid and physical properties of the bounding material. For a tsunami moving through a region of ocean of depth *d*, the assumptions that sea water is incompressible and that viscous forces are negligible lead to the predicted speed *(gd)*^*1/2*^, where *g *is the acceleration due to gravity [1]. For a shock wave moving through an incompressible, non-viscous fluid of density *ρ *in a cylindrical elastic tube of wall thickness *h *and elastic modulus *E*, the speed, denoted *c*_0_, predicted by Korteweg [2] and Moens [3] is

(1) *c*_0_= [(*Eh*)/(2*ρR*)]^1/2^,

The product *Eh *is the ratio of tension in the tube's wall to the fractional amount of circumferential stretching, and *R *is the radius of the tube measured from the central axis to the inner face of the wall.

The speed of the arterial pulse wave is commonly termed the pulse-wave velocity (PWV). It has been shown to increase during the course of certain diseases, and this increase has generally been attributed to "arterial stiffness" [4-7]. This interpretation is consistent with Equation (1), the Korteweg-Moens Equation, which is based in part on the assumption that the fluid is not viscous.

Lambossy [8] introduced a model for arterial blood flow in which viscosity results in shear force on the inner wall of an artery and the pressure gradient is a simple harmonic function of time, $\overset{⌣}{A}$*e*^*iωt*^. In this model, $\overset{⌣}{A}$ is a constant, and *ω *is the frequency of oscillation. Other constants in the model are he viscosity of blood, denoted *μ*, and the density, denoted *ρ*. The arterial wall is a straight, rigid cylindrical tube of radius *R*.

Womersley [9] gave the solution for the Lambossy model. In addition, Womersley incorporated the elastic modulus into the description of the wall of the model artery. Assuming that the change in the tube's radius is small, that the tube is tethered to surrounding structures and that the mass of the tube's wall is negligible, the expression for the PWV for the Lambossy-Womersley model is

(2) $$c^{2}/c_{0}^{2}\approx -J_{2}(i^{3/2}\alpha )/J_{0}(i^{3/2}\alpha ),$$

where *α *= *R*(*ωρ*/*μ*)^1/2^, and *J*_*m*_(*i*^3/2^*α*) is the Bessel function of order *m *and variable *i*^3/2^*α:*

$$\sum_{n=0}^{n=\infty }{\left(-1\right)}^{n}{\left(i^{3/2}\alpha /2\right)}^{m+2n}/[(m+n)!n!].$$

When *α*^2 ^≫ 8, -*J*_2_(*i*^3/2^*α*)/*J*_0_(*i*^3/2^*α*) ≈ 1, and *c*/*c*_0 _is approximately 1. When *α*^2 ^≪ 8, -*J*_2_(*i*^3/2^*α*)/*J*_0_(*i*^3/2^*α*) ≈ *iα*^2^/8, and *c*/*c*_0 _is approximately proportional to *R*. For many years, the result of Womersley has been accepted as a good approximation for the PWV in relatively small arteries where -*J*_2_(*i*^3/2^*α*)/*J*_0_(*i*^3/2^*α*) differs significantly from 1 [10-12].

Some features of arterial blood flow are not well described by the Lambossy-Womersley model. For example, the relative rate of increase in the pressure gradient that results from the power of myocardial contraction is not described accurately by the simple harmonic pressure gradient assumed in the model. Furthermore, Womersley did not incorporate damping of the pressure wave in his model before solving for wave velocity. Womersley also introduced a number of approximations before arriving at the expression for the PWV. Therefore, we investigate the PWV in a model that (1) contains a parameter that describes the relative rate of rise of the pressure gradient, (2) includes an expression for damping of the pressure wave and (3) requires fewer assumptions and approximations in the derivation of an expression for the PWV.

### A model for the leading part of the pulse wave

We start with the solution for the rigid-tube model of Lambossy. A rigorous derivation of the solution for blood volume flow rate, flow velocity and shear force has been published by Painter et al. [13]. The velocity of flow at distance *r *from the central axis of the artery is

$$\overset{⌣}{u}=[\overset{⌣}{A}/(\rho i\omega )]e^{i\omega t}[1-J_{0}(i^{3/2}{\left\{\omega \rho /\mu \right\}}^{1/2}r)/J_{0}(i^{3/2}\alpha )].$$

The volume flow rate is

(3) $$\overset{⌣}{Q}=[\pi \overset{⌣}{A}R^{2}e^{i\omega t}/(i\omega \rho )][-J_{2}(i^{3/2}\alpha )/J_{0}(i^{3/2}\alpha )].$$

We define *I*(*i*^3/2^*α*) = [-*J*_2_(*i*^3/2^*α*)/*J*_0_(*i*^3/2^*α*)]/(*iωρ*) and write

(4) $$\overset{⌣}{Q}=\pi \overset{⌣}{A}R^{2}e^{i\omega t}I(i^{3/2}\alpha ).$$

We define *P*_*Q*_(*i*^3/2^*α*) as *J*_2_(*i*^3/2^*α*)/[(*i*^3/2^*α*)^2^/8] and note that as *R *goes to zero the imaginary part of *P*_*Q*_(*i*^3/2^*α*) vanishes and |*P*_*Q*_(*i*^3/2^*α*)| goes to 1. Substitution now gives

(5) $$\overset{⌣}{Q}=[\pi \overset{⌣}{A}R^{4}/(8\mu )]e^{i\omega t}P_{Q}(i^{3/2}\alpha )/J_{0}(i^{3/2}\alpha ).$$

Therefore, *I*(*i*^3/2^*α*) is approximately *R*^2^/(8*μ*) when *α*^2 ^≪ 8 and is approximately 1/(*iωρ*) when *α*^2 ^≫ 8.

The shear force (per unit area) at the inner wall of the tube is

-*μ *[∂*u*/∂*r*|_*r *= *R *_= -[$\overset{⌣}{A}$*μ*/(*ρiω*)]*e*^*iωt*^*i*^3/2^(*α*/*R*)*J*_-1_(*i*^3/2^*α*)/*J*_0_*(i*^3/2^*α*), where *i*^3/2^(*α*/*R*)*J*_-1_(*i*^3/2^*α*) = *dJ*_0_(*i*^3/2^[*ωρ*/*μ*]^1/2^*r*)/*dr*|_*r *= *R*_. We note that -*J*_-1_(*i*^3/2^*α*) = *J*_1_(*i*^3/2^*α*), which is written as (*i*^3/2^*α*/2)*P*_*S*_(*i*^3/2^*α*). Substitution now gives the expression for the shear force (per unit area), [$\overset{⌣}{A}$*e*^*iωt*^*R*/2]*P*_*S*_(*i*^3/2^*α*)/*J*_0_(*i*^3/2^*α*). This expression is further simplified to

(6) $${-\mu [\partial u/\partial r|}_{r=R}=[\overset{⌣}{A}R/2]e^{i\omega t+i\theta_{PS}-i\theta_{J0}}\left|P_{S}(i^{3/2}\alpha )\right|/\left|J_{0}(i^{3/2}\alpha )\right|.$$

The ratio of shear force per unit length, -2*πRμ*[∂*u*/∂*r*|_*r *= *R*_, to volume flow rate is denoted *K*.

We now replace the harmonic pressure gradient by the exponential gradient *Ae*^*at*^. A solution for this exponential pressure-gradient model is generated by substituting *A *for $\overset{⌣}{A}$ and *a *for *iω *in Equations (4), (5) and (6). Note that, with these substitutions, the Bessel functions become real-valued series in which each term is a positive real number. The solutions for flow rate and shear force per unit area in the exponential pressure wave model are, respectively,

(7) *Q *= [*πAR*^4^/(*8μ*)]*e*^*at*^*P*_*Q*_(*iβR*)/*J*_0_(*iβR*)

and

(8) -*μ*[∂ *u*/∂*r*]|_*r *= *R *_= [*AR*/2]*e*^*at*^*P*_*S*_(*iβR*)/*J*_0_(*iβR*),

where *β *=(*aρ*/*μ*)^1/2^. The function *I*(*iβR*) is equal to *Q *divided by the force gradient *πR*^2^*Ae*^*at*^. When

(9) *aρR*^2^/*μ *<< 8,

*P*_*Q*_(*iβR*), *P*_*S*_(*iβR*) and *J*_0_(*iβR*) are approximately equal to 1, *I*(*iβR*) is approximately *R*^2^/(8*μ*), and the function *K *is approximately equal to 8*μ*/(*ρR*^2^). When *aρR*^2^/*μ *≫ 8, *I*(*iβR*) is approximately equal to 1/(*aρ*), and *K *is approximately equal to 0.

Now consider the fluid motion when the force function, *F *= *πR*^2^*P*, is

*F *= *fe*^*at*^*e*^-*bz*^,

where *a*, *b *and *f *are positive real-valued constants. At time *t*, this expression defines the force function of distance z along the tube. A point defined by a particular value of *z *can be traced backward in time to a point at *z *= 0 on the force function at time *t-z/c*. In the absence of damping as a result of shear forces, the value of the force function would be identical for these two points. Therefore, *fe*^*at*^*e*^-*bz *^= *fe*^*a*(*t*-*z*/*c*)^, so that *b *= *z/c *in the absence of damping.

It is assumed that the ratio of flow rate to force gradient is constant in our model of a segment of an artery that does not contain a branch. An equivalent assumption is that the value of *R*^2 ^does not vary significantly with decreasing pressure along the segment. As a consequence, it follows that flow rat, *Q*, is proportional to *e*^*a*(*t*-*z*/*c*) ^in the absence of damping, *i.e*. the flow wave is described by *Q*_0_*e*^*a*(*t*-*z*/*c*)^, where *Q*_0 _is the flow rate at *t *= 0 and *z *= 0.

In the absence of damping, there is no loss of momentum per unit length in the velocity wave. When there is damping, Equations (7) and (8) imply that the point on the velocity wave at z = 0 at t = 0 loses momentum at specific rate -*K *as it travels distance *z = t/c *in time *t*. Therefore, momentum, velocity and flow rate are reduced by the factor *e*^-*Kz*/*c *^as the flow wave moves a distance equal to *z*, and volume flow rate is proportional to *Q*_0_*e*^*a*(*t*-*z*/*c*) ^*e*^-*Kz*/*c*^. By analogy to the rigid-tube model where flow rate is proportional to the force gradient -∂*F*/∂*z *for small *R*, it is assumed that, in the model where small changes in *R *are allowed, force gradient is also proportional to volume flow rate (for small *R*). Therefore force is likewise proportional to *e*^*a*(*t*-*z*/*c*) ^*e*^-*Kz*/*c*^, and we write

(10) *F *= *fe*^*at*^*e*^-*z*(*a *+ *K*)/*c*^.

The force gradient is

(11) -∂*F*/∂*z *= [(*a *+ *K*)/*c*]*fe*^*at*^*e*^-*z*(*a *+ *K*)/*c*^.

Substitution into Equation (7) gives

(12) *Q *= [(*a *+ *K*)/*c*]*fe*^*at*^*e*^-*z*(*az*+*K*)/*c*^*I*(*iβR*).

For an incompressible fluid in a cylindrical elastic tube, conservation of mass requires that

(13) -∂*Q*/∂*z *= 2*πR*(∂*R*/∂*t*).

Substitution into Equation (12) gives

(14) [(*a *+ *K*)/*c*]^2^*fe*^*at*^*e*^-*z*(*a *+ *K*)/*c *^*I*(*iβR*)- [(*a *+ *K*)/*c*]*fe*^*at*^*e*^-*z*(*az*+*K*)/*c*^(∂*I*(*iβR*)/∂*R*)(∂*R*/∂*z*) = 2*πR*(∂*R*/∂*t*).

For an elastic tube with wall thickness equal to *h *and elastic modulus equal to *E*,

(15) *P *= *Eh*(*R*-*R*_0_)/*R*^2^.

Consequently, *F *= *πEh*(*R *- *R*_0_), and ∂*F*/∂*z *= *πEh*(∂*R*/∂*z*), which is rewritten as

(16) -[(*a *+ *K*)/*c*]*fe*^*at*^*e*^-*z*(*a *+ *K*)/*c *^= *πEh*(∂*R*/∂*z*).

Similarly,

(17) *afe*^*at *^*e*^-*z*(*az*+*K*)/*c *^= *πEh*(∂*R*/∂*t*).

Combining Equations (16) and (17) with Equation (14) gives a description of the flow-pressure coupling (FPC) associated with the pulse wave. The FPC equation will be solved for the PWV. This will be simplified by considering two cases. The first is when *aρR*^2^/*μ *<< 8. *I*(*iβR*) is approximated as *R*^2^(8*μ*), and the function *K *is approximated as 8*μ*/(*ρR*^2^). Combining Equation (16) and (17) with Equation (14) leads to

(18) *Eh*/(2*ρR*) + 2*fe*^*at*^*e*^-*z*(*az*+*K*)/*c*^/(*πρR*^2^) = *c*^2^*aK*/(*a *+ *K*)^2^.

Equation (18) is rewritten as

*Eh*/(2*ρR*)[1 + 4*fe*^*at*^*e*^-*z*(*a *+ *K*)/*c*^/(*πREh*)] = *c*^2^*aK*/(*a *+ *K*)^2^,

and combining this equation with Equations (10) and (15) leads to

(19) *Eh*/(2*ρR*)[1 + 4(*R*-*R*_0_)/*R*] = *c*^2^*aK*/(*a *+ *K*)^2^.

When

(20) 4(*R *- *R*_0_)/*R *≪ 1,

the above equation is approximated as

(21) *c*^2 ^≈ [*Eh*/(2*ρR*)](*a *+ *K*)^2^/(*aK*).

which is rewritten as

(22) $$c^{2}/c_{0}^{2}\approx a/K+2+K/a.$$

Because *a*/*K *= *aρR*^2^/(8*μ*) is assumed to be much smaller than 1,

(23) *c *≈ *c*_0 _[2+8*μ*/(*aρR*^2^)]^1/2^.

This result is not in agreement with Womersley's prediction that the PWV is approximately proportional to *c*_0 _multiplied by *R *when *aρR*^2^/*μ *<< 8.

For the case where *aρR*^2^/*μ *≫ 8, *I*(*iβR*) is approximately equal to 1/(*aρ*), and *K *is approximately equal to 0. Substitution into Equation (14) leads to the Korteweg-Moens expression,

*c*^2 ^≈ *Eh*/(2*ρR*).

The mass of the arterial wall and surrounding tissue was not included in the above analysis. The effect of this mass on the PWV can be assessed by writing a differential equation for its acceleration caused by the difference in fluid pressure in the tube and the force per unit area of the inner wall on the fluid. The solution leads to the approximation

*P *≈ (*a*^2^[*R*-*R*_0_]*h*_*s*_*ρ*_*s *_+ *Eh*)(*R*-*R*_0_)/*R*^2^,

where *h*_*s *_and *ρ*_*s *_are the thickness and density, respectively, of the wall and surrounding tissue accelerated by the increasing arterial pressure.

There are many published estimates of the modulus E for mammalian elastic arteries. Estimates from studies of the change in arterial radius with changes in pressure are usually between 10^6 ^dynes/cm^2 ^and 10^7 ^dynes/cm^2 ^[14,15]. Therefore, the term *a*^2^*R*^2^*h*_*s*_*ρ*_*s *_may be small compared with Eh unless the rate of rise in the arterial pulse is very steep.

### Comparison with Womersley's derivation of the PWV

Womersley [9] replaced the rigid tube of Lambossy with an elastic tube that expands or contracts in response to increasing or decreasing pressure of blood. If the tube is not tethered to surrounding tissue, it also moves axially in response to frictional force of blood on the inner wall. Womersley denoted the radial displacement of the inner wall of the tube by *ξ *and the longitudinal displacement by *ς*. It is assumed that *ξ *and *ς *are described by harmonic functions:

(24) *ξ *= *D*_1 _exp[*iω*(*t *- *z/c*)],

(25) *ς *= *E*_1 _exp [*iω*(*t-z/c*)].

The pressure of the fluid *p *is also assumed to be a harmonic function

(26) *p *= *p*_1 _exp [*iω*(*t-z/c*)].

If *c *is a positive real number, this equation imposes a direction of flow for the pulse wave.

In Equations (35) and (36) of the article by Womersley [9], the boundary conditions for the fluid in contact with the tube's inner wall are described by:

(27) $$i\omega E_{1}=C_{1}+\frac{A_{1}}{\rho_{0}c},$$

(28) $$i\omega D_{1}=\frac{1}{2}\frac{i\omega R}{c}\{C_{1}F_{10}(\alpha )+\frac{A_{1}}{\rho_{0}c}\},$$

where *ρ*_0 _is the density of the fluid, *C*_1 _is a constant and *F*_10_(*α*) is 2*J*_1_(*i*^3/2^*α*)/[*i*^3/2^*αJ*_0_(*i*^3/2^*α*)]. In Equations (37) and (38) of the article, the boundary conditions for the wall of the tube in contact with the fluid are described by:

(29) $$-\omega^{2}D_{1}=\frac{A_{1}}{h\rho }-\frac{B}{\rho }\{\frac{\sigma }{R}(\frac{-i\omega E_{1}}{c})+\frac{D_{1}}{R^{2}}\},$$

(30) $$-\omega^{2}E_{1}=\frac{-\mu }{h\rho R}\{\frac{-C_{1}}{2}i^{3}\alpha^{2}R^{2}F_{10}(\alpha )+\frac{1}{2}\frac{A_{1}}{\rho_{0}c}\omega^{2}\frac{R^{2}}{c^{2}}\}-\frac{B}{\rho }\{\frac{\omega^{2}E_{1}}{c^{2}}+\frac{\sigma }{R}\frac{i\omega D_{1}}{c}\},$$

where *ρ *is the density of the tube, *σ *is Poisson's ratio and *B *is *E*/(1-*σ*^2^).

Womersley interprets Equations (27)–(30) as a system of homogenous linear equations with variables *A*_1_, *C*_1_, *D*_1 _and *E*_1_. Setting the determinant of coefficients equal to 0 gives the equation for *c*. A solution for c is easily found when *σ *= 0 and when the tube is tethered to surrounding structures so that *E*_1 _= 0. Combining Equations (27) and (28) gives

(31) $$D_{1}=-\frac{1}{2}\frac{RC_{1}}{c}\{1-F_{10}(\alpha )\}.$$

Combining Equations (27) and (29) gives

(32) (-*ω*^2^*hρ *+ *Eh/R*^2^)*D*_1 _= -*ρ*_0_*cC*_1_,

and dividing by Equation (31) gives

*c*^2 ^= {-*ω*^2^*Rhρ*/(2*ρ*_0_) + *Eh*/(2*ρ*_0_*R*)}{1-*F*_10_(*α*)}.

Substituting -*J*_2_(*i*^3/2^*α*)/*J*_0_(*i*^3/2^*α*) for 1-*F*_10_(*α*) gives

*c*^2 ^= {-*ω*^2^*Rhρ*/(2*ρ*_0_) + *Eh*/(2*ρ*_0_*R*)}{-*J*_2_(*i*^3/2^*α*)/*J*_0_(*i*^3/2^*α*)}.

Because *ω*^2^*Rhρ*/(2*ρ*_0_) is small compared to *Eh*/(2*ρ*_0_*R*), we have

$$c^{2}/c_{0}^{2}\approx -J_{2}(i^{3/2}\alpha )/J_{0}(i^{3/2}\alpha ).$$

Womersley interprets the imaginary part of 1/*c *multiplied by *ω *as the coefficient in the exponential damping function of the wave as a function of distance. The exponential damping coefficient of time is therefore equal to the real part of *c *times the imaginary part of 1/*c *multiplied by *ω*. When *α*^2 ^<< 8, the real part of *c *is approximately *c*_0_*α*/4, and the imaginary part of 1/*c *is approximately 4/(*c*_0_*α*). Therefore, the technique of Womersley leads to the prediction that the coefficient for the damping with time is approximately equal to *ω *and that this coefficient is approximately independent of the radius in small arteries. This does not make sense because Equations (5) and (6) show that for *α*^2 ^<< 8 the damping coefficient is approximately 8*μ*/(*ρR*^2^), the expression used in the exponential pressure gradient model to describe damping of pulse waves in small arteries.

There is another solution for the PWV in the above equations from the paper of Womersley. Combining equations (27) and (30) for the case where *σ *= 0 and *E*_1 _= 0 leads to

*c*^2 ^= *ω*^2^/[*iα*^2^*F*_10_(*α*)],

which can not be correct because it predicts that *c *does not approach the Korteweg-Moens velocity for large values of *R*.

The equations of Womersley do not contain an expression for the damping caused by shear force between the inner wall of the tube and the moving liquid. If the expression for shear force is added to the Womersley model, Equation (31) becomes

$$i\omega D_{1}=\frac{R}{2}\frac{{\left(i\omega +K\right)}^{2}A_{1}}{i\omega \rho_{0}c^{2}}\{1-F_{10}(\alpha )\},$$

and Equation (32) becomes

(-*ω*^2^*hρ *+ *Eh/R*^2^)*D*_1 _= *iωA*_1_/(*iω*).

When *ω*^2^*ρ *≪ *E*/*R*^2^, the above equations are combined and approximated by

$$c^{2}=\frac{Eh}{2\rho_{0}R}\frac{{\left(i\omega +K\right)}^{2}}{i\omega }\{1-F_{10}(\alpha )\}/(i\omega )$$

When *α*^2 ^<< 8, {1-*F*_10_(*α*)}/(*iω*) is closely approximated by 1/*K*. Furthermore, replacing {1-*F*_10_(*α*)}/(*iω*) by 1/*K *and replacing *iω *by the constant *a *gives Equation (21). Therefore, it appears that the difference between Equation (21) and the corresponding expression derived by Womersely for *c*^2 ^in the tethered, thin-walled tube model is due largely to his omission of the expression for damping due to friction between the wall of the tube and blood moving downstream in the pressure wave.

## Discussion

One possible source of errors in the analysis of this paper is the limited consideration of changes in arterial radius. Such changes are fully considered only when the Law of Laplace, Equation (15), is incorporated in an expression. For this reason, caution should be exercised when using the above results to interpret data from arteries in which there is a considerable change in the radius and the cross-sectional area during the cardiac cycle. In large elastic arteries, this change in radius may be 10% or more from the median value [14], and this may be a source of error that is of concern in certain contexts.

In small arteries where pressure oscillations are of low magnitude, the above concern diminishes. In addition, as arteries become smaller and smaller, the flow becomes closely described by the equations for the rigid tube. Consequently, the damping function approaches the damping function calculated from Poiseuille's Equation. Therefore, concern for errors in the analysis is less for the expression giving the PWV in small arteries than it is for the expression giving the PWV in large arteries.

A plausible explanation for an increase in the PWV during the course of a disease is an increase in arterial stiffness leading to an increase in the parameter *E*. This explanation is largely based on the Korteweg-Moens equation. An increase in the elastic modulus, *E*, or the relative thickness of the arterial wall, *h/R*, would increase the PWV. However, changes in other properties associated with arterial walls and surrounding tissue may also increase the PWV and may be interpreted as increasing stiffness. One source of arterial stiffness that is not considered in the above analysis is production of heat as the arterial wall is stretched [14].

The above results show that the PWV can increase in small arteries if the parameter *a *decreases. The parameter *a *describes the rate of increase in pressure during the initial rise as the pulse wave approaches a point in an artery. This rise is determined by the rise in the ejection rate through the aortic valve in early systole. A number of heart disorders can affect this rate of rise. Examples include aortic stenosis, myocardial ischemia and certain conduction disorders. It appears plausible that, in certain diseases of the heart, attributing an increase in the PWV to arterial stiffness may not be the correct explanation. The link between an increase in the PWV and increased risk of myocardial infarction [7] may be due, at least in part, to myocardial ischemia.

## Competing interests

The author declares that they have no competing interests.
